# Delimiting Coalescence Genes (C-Genes) in Phylogenomic Data Sets

**DOI:** 10.3390/genes9030123

**Published:** 2018-02-26

**Authors:** Mark S. Springer, John Gatesy

**Affiliations:** 1 Department of Evolution, Ecology, and Organismal Biology, University of California, Riverside, CA 92521, USA; 2 Division of Vertebrate Zoology and Sackler Institute for Comparative Genomics, American Museum of Natural History, New York, NY 10024, USA

**Keywords:** coalescence genes, phylogenomics, protein-coding sequences, recombination breakpoints, recombination ratchet

## Abstract

Summary coalescence methods have emerged as a popular alternative for inferring species trees with large genomic datasets, because these methods explicitly account for incomplete lineage sorting. However, statistical consistency of summary coalescence methods is not guaranteed unless several model assumptions are true, including the critical assumption that recombination occurs freely among but not within coalescence genes (c-genes), which are the fundamental units of analysis for these methods. Each c-gene has a single branching history, and large sets of these independent gene histories should be the input for genome-scale coalescence estimates of phylogeny. By contrast, numerous studies have reported the results of coalescence analyses in which complete protein-coding sequences are treated as c-genes even though exons for these loci can span more than a megabase of DNA. Empirical estimates of recombination breakpoints suggest that c-genes may be much shorter, especially when large clades with many species are the focus of analysis. Although this idea has been challenged recently in the literature, the inverse relationship between c-gene size and increased taxon sampling in a dataset—the ‘recombination ratchet’—is a fundamental property of c-genes. For taxonomic groups characterized by genes with long intron sequences, complete protein-coding sequences are likely not valid c-genes and are inappropriate units of analysis for summary coalescence methods unless they occur in recombination deserts that are devoid of incomplete lineage sorting (ILS). Finally, it has been argued that coalescence methods are robust when the no-recombination within loci assumption is violated, but recombination must matter at some scale because ILS, a by-product of recombination, is the raison d’etre for coalescence methods. That is, extensive recombination is required to yield the large number of independently segregating c-genes used to infer a species tree. If coalescent methods are powerful enough to infer the correct species tree for difficult phylogenetic problems in the anomaly zone, where concatenation is expected to fail because of ILS, then there should be a decreasing probability of inferring the correct species tree using longer loci with many intralocus recombination breakpoints (i.e., increased levels of concatenation).

## 1. Introduction

The nature of ‘characters’ is fundamentally important to systematics and has attracted the attention of researchers for several decades [1,2,3,4,5]. Characters are usually assumed to be independent of each other, so that changes in one character are not correlated with changes in another character. For example, changes in an upper molar may be independent of changes in fur color. By contrast, changes in the size of the protocone, which is one of the primary cusps on each upper molar of a typical mammalian tooth, are expected to be functionally correlated with the size of the talonid valley, which is the basin on the corresponding lower molar that occludes with the protocone when the jaws are closed. At the molecular level, ribosomal and transfer RNA genes provide compelling cases of dependence because of the base pairing between sites that is favored in the stem (helical) regions of these molecules [6].

Independence at the genetic level is also constrained by linkage relationships [2]. More specifically, different segments of a chromosome can have different genealogical histories because of incomplete lineage sorting (ILS) and the retention of ancestral polymorphisms (deep coalescence). A coalescence gene, or c-gene [2], is “a segment of the genome for which there has been no recombination over the phylogenetic history of a clade” ([7], p. 33). C-genes are separated from adjacent c-genes by recombination breakpoints that mark the boundaries of these units. C-genes are the basic unit of analysis for most coalescence methods that are used to infer species trees with summary coalescence methods such as STAR [8], MP-EST [9], NJst [10], and ASTRAL-II [11]. Indeed, a basic assumption of these coalescence methods is that recombination occurs freely among c-genes but not within c-genes. When sequence evolution is completely neutral, c-genes and their associated genealogical histories (trees) are expected to occur with frequencies that can be predicted by the multispecies coalescent (MSC) given a species tree with its constituent branch lengths (in coalescent units). The quantitative relationship between the frequencies of different gene tree topologies and the species tree has been dubbed gene tree stoichiometry [7]. If the stoichiometry of observed gene trees accurately reflects the expected stoichiometry of gene trees under the MSC, then summary coalescence methods will converge on the true species tree given enough gene trees that are randomly sampled from throughout the genome [12]. However, altered gene tree stoichiometry can lead to a different species tree just as different stoichiometries for the reactants in a chemical equation can yield different chemical products. It is therefore essential to employ gene trees that reflect their true frequencies if the objective is to infer a species tree with accurate topological relationships and branch lengths (in coalescence units) [7]. A problem arises, however, because if c-genes are too short, they will not contain enough phylogenetic signal to accurately resolve gene tree topologies and proper stoichiometry will be impacted. At the other extreme, if c-genes are artificially too long and are instead “pseudo” c-genes that encompass intralocus recombination breakpoints, then stoichiometry again will be distorted. The usage of pseudo c-genes in phylogenetic coalescence analysis, ‘concatalescence’ [13], will have a tendency to shift gene tree stoichiometry towards the most common gene tree, and potentially prevent coalescence methods from addressing problems with ILS in the anomaly zone where the species tree is different from the most common gene tree [7]. One approach to avoid the c-gene conundrum is through the use of SNP methods, which ideally use variation at widely spaced, independently segregating, individual nucleotide positions. For example, Chifman and Kubatko’s [14] SVDquartets method uses SNP variation to infer quartet trees that are then amalgamated into a species tree. However, issues associated with c-gene size remain front and center for other coalescence methods or when closely linked nucleotide sites are analyzed using SNP methods such as SVDquartets that assume independence of each SNP in the analysis.

Here, we review some of these issues that are central to proper application of summary coalescence methods to genome-scale data by focusing on key papers in the field that have recently been published. In the context of our review we also rebut specific criticisms of scientists [15,16,17] who have misinterpreted our earlier work [7]. We begin with a detailed description of the recombination ratchet (Section 2) and show that when recombination is present, c-genes generally become smaller as more taxa are added to a phylogenetic study. In Section 3, we address criticisms of the recombination ratchet [15] that misrepresent this concept and have been perpetuated in the literature [16,17]. Section 4 provides a review of the science that underpins estimates of c-gene size including applications of a coalescent hidden Markov model (CoalHMM) [18] to four taxa with extrapolations to larger data sets using the recombination ratchet [7]. This section also reviews a recent empirical study [19] that addresses whether or not exons from the same gene share a common branching history, or alternatively, several conflicting topologies. Section 4 corrects previous misattributions of Edwards et al. [15] to our earlier work [7]. In Section 5, we ask whether complete protein-coding sequences are appropriate for coalescence analyses. Section 6 reviews two simulation studies [20,21] that ask whether recombination is a problem for summary coalescence methods. We conclude that recombination must matter at some scale if coalescence methods effectively address the problem of ILS. We also call attention to the need for simulation studies that incorporate recombination at deep phylogenetic levels and compare summary coalescence methods versus concatenation. In Section 7, we compare different recombination detection methods and conclude that CoalHMM infers more recombination breakpoints than other approaches. We also demonstrate the importance of using contiguous sequences, including the intervening introns, when recombination detection methods are applied to complete protein coding sequences. In Section 8, we evaluate the use of summary coalescence methods with binned versus transcriptome data and conclude that previous criticisms of binning [22] also apply to transcriptome data. Section 9 summarizes our major findings and outlines critical issues for moving forward with summary coalescence methods.

## 2. The Recombination Ratchet

**Figure 1:**
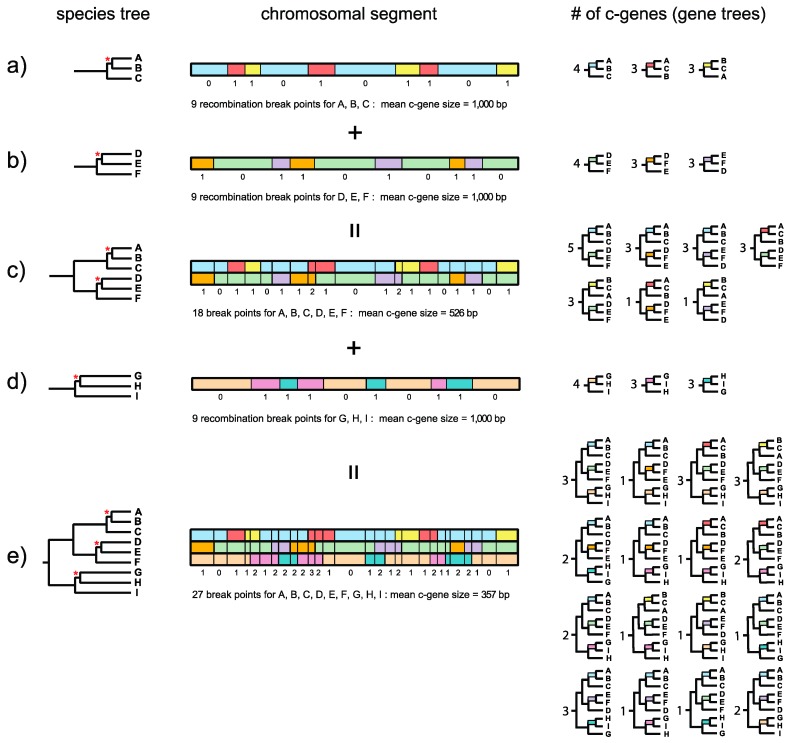
Illustration of the recombination ratchet for a hypothetical 10-kb chromosomal segment of DNA and a phylogeny with nine taxa and three short internodes, each of which results in incomplete lineage sorting and deep coalescence for a local subtree with three taxa. The three short internodes (internal branches) are labeled with red asterisks. Other internal branches are longer, and deep coalescence does not occur. The three subtrees are for taxa A-B-C (**a**); D-E-F (**b**); and G-H-I (**d**). Incomplete lineage sorting for each set of three taxa (**a**,**b**,**d**) is associated with nine recombination breakpoints and ten c-genes of average length 1000 bp. For each set of three taxa there are three different genealogical histories with contrasting topological relationships (genealogical histories for the same topology but with different branch lengths are ignored). Numbers below c-genes correspond to Robinson–Foulds (RF) distances [24], *sensu* Sul and Williams [25], relative to the species tree. Note that for each chromosomal segment with only three taxa (panels **a**,**b**,**d**), the maximum RF distance is 1. The overlay of nine recombination breakpoints for A-B-C and nine recombination breakpoints for D-E-F results in a total of 18 recombination breakpoints and 19 c-genes for the six-taxon phylogeny (A-B-C-D-E-F) (panel **c**). Average c-gene size for the 10-kb chromosomal segment with six-taxa is 526 bp. Nine different topologies are possible for these six taxa, of which seven are represented among the 19 c-genes. The maximum RF distance is 2 for c-genes based on six taxa (panel **c**). The overlay of 18 recombination breakpoints for A-B-C-D-E-F with nine recombination breakpoints for G-H-I results in 27 recombination breakpoints and 28 c-genes for the nine-taxon phylogeny (A-B-C-D-E-F-G-H-I) (panel **e**). For the nine-taxon phylogeny, mean c-gene size is reduced to 357 bp. Among the 28 c-genes for the nine-taxon phylogeny, 16 of 27 possible topologies are represented (panel **e**).

Dutheil and Hobolth [23] illustrated the relationship between recombination breakpoints, topology, and genealogical changes along a chromosomal segment for a three-taxon problem with human, chimp, and gorilla. The recombination ratchet with its connection to the genealogical histories of adjacent c-genes is illustrated in Figure 1 and is a logical extension of this framework for estimating c-gene size when there are more than three taxa. Springer and Gatesy ([7], p. 33) defined the recombination ratchet as “the inverse relationship between c-gene size and the number of taxa that is driven by recombination in different regions of a phylogenetic tree.” Suppose that a species tree for three taxa (A, B, C) has a very short internal branch (in coalescence units) that unites A and B to the exclusion of C (Figure 1a). Because this internal branch is very short and is susceptible to ILS, not all gene trees will agree with the species tree. Instead, chromosomal segments will be comprised of adjacent c-genes that support each of three different topologies, i.e., A + B, A + C, and B + C. Boundaries between these c-genes correspond to recombination breakpoints. In the example in Figure 1a there are nine recombination break points along a 10-kb chromosomal segment so that there are 10 c-genes with a mean c-gene size of 1000 bp. Four c-genes support A + B, three c-genes support A + C, and three c-genes support B + C (Figure 1a). The Robinson–Foulds (RF) distances [24] for individual c-gene trees (relative to the species tree) are either 0 or 1 for a three-taxon species tree such as ((A,B),C) with one short internal branch when all c-gene trees are bifurcating. (Note that RF distances are *sensu* Sul and Williams [25] and are equivalent to RF/2 of Robinson and Foulds [24].).

Now suppose that a second species tree for three taxa (D, E, F) also has a very short internal branch (Figure 1b). In this case the short branch unites D and E to the exclusion of F. As for the first species tree, not all gene trees will agree with the species tree and there will be c-genes that support D + E, D + F, and E + F. If the orthologous 10-kb chromosomal segment for these three taxa also shows nine recombination breakpoints then there again will be 10 c-genes with a mean c-gene size of 1000 bp (Figure 1b). In this hypothetical example, four c-genes support D + E, three c-genes support D + F, and three c-genes support D + F (Figure 1b). Importantly, recombination breakpoints for the A-B-C and D-E-F species trees are independent of each other, so that there are a total of 18 different recombination breakpoints. Now suppose that we combine these two 3-taxon species trees into a single species tree for all six taxa (A, B, C, D, E, F) (Figure 1c). Further suppose that the internal branches leading to A-B-C and to D-E-F are both long and therefore insulated from ILS deeper in the tree. The resulting species tree for six taxa, with its two very short internal branches, will therefore allow for nine different topologies, i.e., three resolutions of A-B-C multiplied by three resolutions of D-E-F. In our example, 7 of 9 possible c-genes (and associated gene trees) are represented in the 10-kb chromosomal segment (Figure 1c). Because the total number of recombination breakpoints is now 18 for this 6-taxon species tree, the total number of c-genes increases to 19 and mean c-gene size shrinks from 1000 bp to 526 bp (Figure 1c). Also note that the maximum RF distance between a gene tree and the species tree increases from 1 for a three-taxon species tree with one short internal branch to 2 for a six-taxon species tree with two short internal branches.

Finally, consider a third species tree for the three taxa G, H, and I (Figure 1d). This tree also has a very short internal branch that unites G and H to the exclusion of I. As for the A-B-C and D-E-F three-taxon species trees, not all gene trees will agree with the species tree. Instead, some trees will support G + H, some will support G + I, and still others will support H + I. Also, suppose that the orthologous 10-kb chromosomal segment has nine recombination breakpoints as for the other two three-taxon species trees (A-B-C, D-E-F). When this 10-kb chromosomal segment is considered in isolation for G-H-I, there again will be 10 c-genes with a mean size of 1000 bp (Figure 1d). However, the recombination break points for G-H-I are independent of the recombination break points for A-B-C and D-E-F, so that when A-B-C, D-E-F, and G-H-I are all combined into a nine-taxon species tree there will be 27 recombination break points and 28 c-genes with a mean length of only 357 bp (Figure 1e). Note that this calculation ignores the possibility of recurrent (homoplastic) recombination breakpoints at the exact same position in the gene. If we assume that A-B-C-D-E-F and G-H-I both have long stem branches, then ILS will be constrained to the three very short branches that occur within A-B-C, D-E-F, and G-H-I, respectively. If we ignore branch length heterogeneity [26], the net result is that 27 different c-genes (and associated gene trees) are possible, i.e., three resolutions of A-B-C multiplied by three resolutions of D-E-F multiplied by three resolutions of G-H-I. Among the 27 possible c-gene topologies that are theoretically possible, 16 are represented in Figure 1e. Importantly, this example shows that c-genes become smaller as more species are added to a phylogenetic study. The effects of the recombination ratchet on c-gene size can be diminished by analyzing data sets with only a few taxa (e.g., three taxa and an outgroup), but phylogenetic analyses with a limited number of taxa have their own attendant problems (e.g., long branch misplacement in analyses where rates among lineages differ and divergence is great) and are insufficient for analyzing rapid radiations where difficult to resolve polytomies are associated with two or more consecutive short internal branches.

Figure 1e also shows the RF distance of each gene tree relative to the species tree. The gene tree on the left has an RF distance of 1 relative to the species tree. Individual gene trees in Figure 1e have RF distances that range from 0 to 3, where the maximum value of 3 is constrained by the number of short internodes (3) that allow for ILS. Trees for adjacent c-genes have RF distances that are either equal to each other or differ by a value of 1. This is because gene tree topologies are autocorrelated when there is more than one short internal branch that permits ILS. By contrast, RF distances will not be autocorrelated when there is only one trichotomy because the maximum RF distance between gene trees and the species tree, or between adjacent gene trees, is 1 (Figure 1b). Autocorrelation poses an additional problem for summary coalescence analyses because these methods assume that c-genes are independent of each other.

## 3. Criticisms of the Recombination Ratchet

Edwards et al. ([15], p. 448) suggest that the recombination ratchet is flawed and causes “more confusion than clarity in thinking about phylogenetic data.” Later, Edwards et al. ([15], p. 452) state that they “view recombination breakpoints as an intrinsic property of individual species, not entire clades, and thus adding taxa to a problem involving recombination does not make sense.” This interpretation misrepresents our original text [7]. We agree that recombination breakpoints in one part of the species tree (e.g., three species of *Equus* [horses]) are independent of recombination breakpoints in another part of the tree (e.g., human-chimp-gorilla clade). If a coalescence analysis is limited to human, chimp, and gorilla, and if a given locus shows no recombination breakpoints for these three taxa, then it is appropriate to treat this locus as a single c-gene; we have never said otherwise. However, if a phylogenetic coalescence analysis includes the three hominid primates plus three equids, and if a given locus shows a recombination breakpoint for the three equids, then it is inappropriate to treat this locus as a single c-gene because this locus has two different branching histories when the full tree of six taxa is considered. Instead, a coalescence analysis that includes the three hominid species and the three species of *Equus* should divide this locus into two separate c-genes because of the recombination breakpoint in *Equus*; failure to recognize this breakpoint would violate an assumption of many summary coalescence methods—no intralocus recombination [8]. There is also an autocorrelation problem due to close linkage (i.e., c-genes are adjacent) and ideally just one of the two c-genes should be utilized in a coalescence analysis. The inclusion of both of these adjacent c-genes would violate another assumption of these summary coalescence methods—free recombination between loci [8]. It may be tempting to choose the longer of the two c-genes, which on average might contain more informative variation, but this choice could bias the analysis given that there may be size differences between c-genes that support different topologies [18].

Edwards et al.’s [15] misinterpretation of the recombination ratchet has been perpetuated in subsequent studies [16,17]. For example, Xu and Yang ([16] p. 1356) state that “for a eutherian mammal dataset, Springer and Gatesy (2016) used empirical estimates of primate recombination rates to calculate the c-gene size to be ~12 bp. However, this calculation is unnecessarily stringent. All sites at the locus will have the same gene tree topology and coalescent times so that the MSC density of Rannala and Yang (2003) **will be valid as long as there is no recombination during the parts of the gene tree where coalescent events occur** (Lanier and Knowles 2012; Edwards et al. 2016).” The bolded section of this quote is precisely the point of the recombination ratchet: recombination does occur in other parts of the gene tree where coalescent events occur. All sites in a particular locus may have the same gene tree topology and coalescent times for one part of the tree (coalescent event), but if these sites have different topologies and branch lengths in other parts of the tree where there are independent coalescent events then it is not appropriate to treat this locus as a single c-gene for all taxa in the phylogenetic analysis. If a gene tree is inferred from a locus that has recombination breakpoints for some of the constituent taxa, then the inferred tree will index multiple, mixed genealogies.

By contrast with Edwards et al.’s [15] dismissal of the recombination ratchet, the multispecies coalescent recognizes the distinct history of each c-gene even though recombination events that affect genealogical histories in one lineage (human-chimp-gorilla) are unrelated to recombination breakpoints that affect a different lineage (three species of *Equus*). Coalescence programs for simulating gene trees assume that individual gene trees, and their underlying c-genes, have genealogical histories that are the same for the entire alignment (i.e., there are no recombination breakpoints anywhere in the alignment for a given c-gene). Thus, there is a fundamental disconnect between c-gene boundaries in simulation studies, where exact c-genes are usually known and do not have mixed histories, versus most empirical studies that are recombination-ignorant and simply assume no recombination without any justification or analysis to back up the assertion.

The assumption of no recombination in empirical studies is most problematic when scientists employ complete protein-coding sequences for a gene that are stitched together from distantly located exons that can be as far apart as a megabase or more [7,13]. Such sequences are also utilized in phylogenomic analyses of transcriptomes. A recent empirical study suggests that in mammals, discontiguous exons from the same gene have genealogical histories that are just as different from each other as are those of exons from different genes, and therefore should not be merged for usage in summary coalescence analyses [19]. Instead of employing a recombination-ignorant approach, it could be profitable to estimate/determine recombination breakpoints and address potential problems with autocorrelated c-genes before analyzing data with summary coalescence methods. Otherwise gene tree stoichiometry will be distorted, and theoretical guarantees for the superiority of the multispecies coalescent are null and void. Unfortunately, recombination breakpoints are difficult to determine, especially when they delineate c-genes that support the same topology and only differ because of branch length heterogeneity [18]. Similarly, recombination breakpoints may be difficult to determine with increasing taxonomic diversity because of the inverse relationship between c-gene size and the number of taxa. Smaller and smaller c-genes that result from the recombination ratchet have progressively less information for resolving relationships across the entire tree with more and more taxa. Also, recombination breakpoints and ILS that are associated with increasingly divergent taxa will exhibit higher levels of homoplasy. Still, coalescence analyses will be compromised if the critical issue of c-gene boundaries is simply ignored [21]. One strategy may be to search for recombination breakpoints with small subsets of taxa that are associated with individual coalescent events (e.g., human-chimp-gorilla or three species of *Equus*) and then to combine these together to produce a c-gene map for the full set of taxa. Large data sets with numerous short branches could also be analyzed iteratively with c-gene boundaries that are limited to specific parts of the tree if the goal is to resolve local polytomies one at a time. For example, analyses with c-gene boundaries that are specific for human-chimp-gorilla or for the three species of *Equus* could be performed separately with the full data set and then combined into the same species tree. However, if the goal is to simultaneously resolve polytomies across the entire tree, then it is not appropriate to only consider recombination breakpoints for one of the local polytomies.

Ideally, attempts at determining recombination breakpoints for complete coding sequences should be based on alignments that include coding exons as well as all of the intervening introns. The reason for including coding sequences and intervening introns can be illustrated with a simple example. Suppose that exon 1 and exon 2 for a given gene both support ((human, chimp), gorilla) and appear to represent one history when only coding sequences are analyzed. However, if intron 1 has an intervening segment that supports ((human, gorilla), chimp), then exon 1 and exon 2 are separate c-genes and should be treated accordingly to satisfy the multispecies coalescent and maximize the integrity of gene tree stoichiometry.

## 4. How Big Are C-Genes?

Hobolth et al. [18] employed a coalescent hidden Markov model (CoalHMM) to estimate recombination breakpoints and the mean length of fragments that support alternate resolutions of the human–chimp–gorilla trichotomy. Four autosomal regions of the genome were examined, which they referred to as targets 1, 106, 121, and 122. Hobolth et al. [18] recognized four resolutions of the trichotomy: human + chimp without deep coalescence (HC1), human + chimp with deep coalescence (HC2), chimp + gorilla (CG), and human + gorilla (HG). After applying their CoalHMM model to the four genomic targets, Hobolth et al. [18] concluded that the mean length of fragments that support HC1 range from 532 to 2710 bp for these four genomic regions, whereas the estimated mean lengths are much shorter for the three other states and have mean lengths that range from 41 to 81 bp. Hobolth et al. [18] also estimated the proportions of each autosomal region that reside in each of the four states (i.e., HC1, HC2, CG, HG), which resulted in mean c-gene sizes for all four states combined that range from 84 to 123 bp for Hobolth et al.’s [18] four autosomal regions.

Springer and Gatesy [7] extrapolated these calculations to regions of the genome that are 139.6 kb, which is the mean length of 447 loci from Song et al. [27] from start codon to stop codon. Springer and Gatesy’s [7] calculations were also extrapolated to demonstrate the effects of the recombination ratchet on c-gene size for a mammalian species tree with nine short internodes as in Song et al.’s [27] mammalian species tree for 37 taxa. Springer and Gatesy [7] estimated that mean c-gene length will shrink to ~12 bp when there are nine short internodes that are similar to the internal branch that joins human and chimp to the exclusion of gorilla.

Edwards et al. ([15], p. 452) found our “conclusion that 12-bp defines the largest length of DNA that is suitable for phylogenetic analysis (p. 29) unsubstantiated.” First, Springer and Gatesy [7] never concluded that 12 bp is the largest length of DNA that is suitable for phylogenetic analysis. Instead, we suggested that mean c-gene size may be on the order of ~12 bp for Song et al.’s [27] dataset for 37 taxa with ~9 short internodes if basing our estimate on c-gene sizes reported by Hobolth et al. [18]. The size of c-genes in a given data set will vary with taxon sampling, recombination rates, etc. In general, c-genes will be smaller with more taxa and larger with fewer taxa as a consequence of the recombination ratchet, which dictates an inverse relationship between the number of taxa and c-gene size. Also, individual c-genes will vary in length depending on the locations of recombination breakpoints. This is evident from Hobolth et al.’s [18,28] estimates of c-gene size for hominid primates, which show that the distribution of fragment lengths for each state (e.g., human + gorilla) is approximated by the geometric distribution. On a broader scale, different regions of the genome range from “recombination deserts” where recombination rates are low to “recombination jungles” where recombination rates are high [29,30].

Edwards et al. [15] also challenged our estimate of c-gene size based on Hobolth et al. [18]: “Depending on the scale over which Hobolth et al. (2007) applied their algorithm, they often estimated much longer tracts of topologically homogeneous DNA, sometimes on the order of tens of kilobases (e.g., their Figure 3), than the 109 bp in primate Tract 122 that Springer and Gatesy (2015) highlight.” Here, Edwards et al.’s [15] argument in favor of longer c-genes is mistakenly based on maximum c-gene size rather than mean c-gene size. Hobolth et al.’s [18] Figure 3 is based on “Target 1” and does show individual tracts that in some cases are several kilobases in length. However, the mean size of c-genes in this target region is only slightly larger (~123 bp) than the mean size (~109 bp) of c-genes in Target 122 (see Table 1 in Springer and Gatesy [7]). Indeed, two of Hobolth et al.’s [18] other target regions have mean c-gene sizes that are only ~84 and ~103 bp, respectively [7].

Finally, Edwards et al. [15] suggest that we “mistakenly attribute homoplasy across the mammal tree with recombination, thereby invalidating” our arguments. This attribution is false. Springer and Gatesy’s [7] estimates of c-gene size were based on values that were calculated by Hobolth et al. [18] with a coalescent hidden Markov model (CoalHMM). CoalHMM models do not equate homoplasy with recombination and are more complex than other approaches (“independent loci”, “site patterns”) [23] that are sometimes used to estimate recombination breakpoints. Hobolth et al.’s [18] model was designed to analyze a four species alignment that includes three ingroup taxa (human, chimp, gorilla) and one outgroup (orangutan). Hobolth et al.’s [18] model allows for four hidden states, which represent four genealogies but only three topologies because the two most closely related taxa (e.g., human and chimp) may coalesce in their immediate ancestral population (HC1 of Hobolth et al. [18]) or in the population that is ancestral to all three ingroup species (HC2 of Hobolth et al. [18]). Hobolth et al.’s [18] model includes transition probabilities between genealogies along chromosomal segments and explicitly accounts for local rates of substitution, branch lengths, and recurrent mutations. The likelihood of a particular genealogy is summed over all possible segmentations. CoalHMM models have been widely used to infer speciation times, patterns of gene flow, changes in population size, and recombination rates [18,28,31,32,33,34,35,36,37]. Hobolth et al. [18] also found that filtering out hypervariable CpG sites had minimal impact on their results.

More recently, Munch et al. [38] employed CoalHMM to infer 1,059,537 recombination events over ~2 GB of the genome in the common ancestor of human and chimpanzee, which suggests that genome-wide estimates of mean c-gene size are approximately 19× larger than estimated by Hobolth et al. [18], i.e., closer to 1.9 kb than 0.1 kb as estimated by Hobolth et al. [18] for targeted autosomal regions. Still, even with this much larger estimate, extrapolation with the recombination ratchet reduces mean c-gene size to ~19 × 12 bp = ~228 bp for a phylogeny with 36 mammals and nine short internodes as in Song et al. [27].

By contrast with Hobolth et al.’s [18] model-based Bayesian coalescence approach (CoalHMM), Edwards et al. [15] suggest that c-genes can be much longer based on White et al.’s [39] analysis of *Mus* genome sequences for three subspecies of the house mouse, *Mus musculus musculus*, *M. m. castaneus*, and *M. m. domesticus*. White et al. [39] reported c-genes with an average length of ~98.2 kb. However, White et al. [39] started with fragments that are 100 SNPs in length, where the mean length of 100 SNPs is ~42.5 kb (C. Ané, pers. comm. to J.G.), and then combined adjacent fragments into larger fragments when phylogenetic incongruence between adjacent fragments was below a specified threshold as measured by the minimum description length (MDL) principle. Unlike Hobolth et al.’s [18] Bayesian method, the MDL approach is based explicitly on a penalty for homoplasy, the value of which is arbitrary. White et al.’s [39] estimate of a mean size of 98.2 kb across the genome was based on their maximum penalty of 3. However, when the minimum penalty of 0.9039 was applied to chromosomes 18, 19, and X, the number of loci on each chromosome roughly doubled, and locus size was cut in half. Half of 98.2 is only 49.1, so that White et al.’s [39] procedure only resulted in a minimal increase in locus size given a starting size of ~42.5 kb. In summary, White et al.’s [39] approach started with c-genes that were potentially much too long, in part because of computational demands, and then blended these c-genes together with a coarse-grained, homoplasy-based method that necessarily has reduced sensitivity to the signal in the primary data (i.e., individual SNPs). Importantly, this method was not used to detect c-genes that are shorter than 100 SNPs in length (i.e., ~42.5 kb) and is much less sensitive than the coalescence approach (CoalHMM) employed by Hobolth et al. [18,28]. Edwards et al. [15] mistakenly suggested that we equated homoplasy with recombination even though we explicitly cited the Bayesian model-based CoalHMM multiple times in our discussion [7], but ironically Edwards et al. [15] conflated homoplasy with recombination. Similarly, Song et al. ([27], p. 14945) equated homoplasy with recombination when they “tested for the effect of recombination by plotting the consistency index of loci, a measure of homoplasy and hence recombination, versus the length of each locus.”

Most recently, Scornavacca and Galtier [19] examined 7349 distinct mammalian coding exons that were downloaded from OrthoMaM v9 [40] and included sequences for 39 placentals and three marsupials. Scornavacca and Galtier [19] reconstructed gene trees for each locus with RAxML and then assessed whether exons from the same gene share a common, branching history of coalescence that is different from exons carried by other genes. Scornavacca and Galtier [19] used RF distance as a metric for these comparisons, and found that there were no differences between exons belonging to the same gene and exons from different genes. These results support the conclusion that separate exons from the same gene do not share a common genealogy. Based on these results, Scornavacca and Galiter [19] also concluded that ILS was only a minor determinant of gene tree heterogeneity for the mammalian exons that they analyzed. Beyond these analyses, the authors performed similar analyses but with half exons rather than full exons, and determined that half exons belonging to the same exon have more similar histories than do half exons that belong to different exons. At the same time, this result does not demonstrate that half exons belonging to the same exon have the same history. This is because half exons belonging to the same exon may be comprised of distinct c-genes that are nevertheless more similar to each other than to half exons belonging to other exons because of autocorrelation between adjacent c-genes (see Figure 1e).

## 5. Are Complete Protein-Coding Sequences Appropriate for Coalescence Analyses?

Zhong et al. [41] defended the use of complete protein-coding sequences in coalescence studies, as occurs with transcriptome data, because “combining the fragmental sequences from the same gene into single protein-coding loci has been widely used in phylogenetic studies.” Edwards et al. [15] also defended the use of transcriptome data with coalescence methods based on the frequency of this practice among systematists and broad congruence with studies that employed concatenation. However, this appeal to the widespread use of transcriptome data in coalescence studies is an *argumentum ad populum*, an informal fallacy in logic. Equating success by general agreement with an alternative method that does not account for ILS also is not a particularly compelling argument because coalescence and concatenation methods often disagree at contentious nodes (the ones where phylogenomic data are often brought to bear) (e.g., [41]). Song et al.’s [27] original defense for using longer loci in coalescence studies was based on an analysis where they tested for the effects of recombination by plotting the consistency index of each locus, a measure of homoplasy and by proxy recombination, versus the length of each locus. The expectation was that there should be a positive correlation if recombination is a systematically confounding factor. To their credit, and by contrast with other studies that have employed transcriptome data or complete protein-coding sequences extracted from genomes [42,43,44,45,46,47,48,49,50,51,52], Song et al. [27] recognized that recombination is a potentially confounding factor in coalescence studies with longer loci. Unfortunately, Song et al.’s [27] analyses assumed an average locus size of 3.1 kb because their calculations did not account for intervening introns in their protein-coding sequences. Instead, the average locus size from start codon to stop is 139.6 kb with some loci exceeding one million bp. The result of this miscalculation is that Song et al.’s [27] conclusion that “recombination appears not to be a systematically confounding factor in this data set” is invalid [13,53]. By contrast with Edwards et al.’s [15] assertion that it is appropriate to use complete protein-coding sequences with summary coalescence methods, Springer an Gatesy’s [7] theoretical calculations based on the recombination ratchet suggest c-genes are smaller by several orders of magnitude than are the c-genes that were employed by Song et al. [27]. Scornavacca and Galtier’s [19] results provide additional support for this conclusion and suggest that different exons belonging to the same gene have different histories just as exons from different genes have different histories. Scornavacca and Galtier’s [19] results also suggest that individual exons are more appropriate for analyses with summary coalescence methods than are complete protein-coding sequences, but even the former may be comprised of multiple c-genes.

Beyond Song et al.’s [27] discredited calculations that ignored the presence of introns and Edwards et al.’s [15] appeal to the widespread use of summary coalescence methods with complete protein-coding sequences, Edwards et al. [15] also cited a simulation study by Lanier and Knowles [20] as justification for using summary coalescence methods even when there is recombination within loci. As discussed below, Lanier and Knowles’s [20] simulations are of little or no relevance to phylogenomic analysis of deep problems in the Tree of Life (the primary focus of our critiques) because these authors simulated shallow divergences for only a handful of loci and a few taxa, and provided no results on the performance of coalescence versus concatenation methods for their simulated data.

## 6. Simulations of Coalescence with Recombination

Lanier and Knowles [20] simulated recombination for neutrally evolving sequences that included one, three, or nine genes, each of which comprised 1000 bp and eight taxa. They analyzed these data with two coalescence methods, a fully Bayesian approach (*BEAST) and a likelihood method (STEM). Most of Lanier and Knowles’ [20] simulations were at total tree depths of 1 N, 2 N, 4 N, and 10 N, although they also performed simulations of “deep radiations” with a total tree depth of 20 N. Given maximum tree depths of ~0.03 substitutions per site for their 10 N simulations (their Figure 7), the total tree depth for their maximum depth trees (20 N) should be ~0.06 substitutions per site, which is equivalent to ~27 million years given a neutral rate of evolution of 2.2 × 10^3^ substitutions/site/million years for mammalian genomes [54]. Thus, levels of sequence divergence for neutrally evolving loci in Lanier and Knowles [20] appear to be well matched to interspecific divergences in the Plio-Pleistocene (1 N) through deeper divergences that extend into the Oligocene (at least for mammals). However, these simulations are less well matched to deeper phylogenetic problems such as the placental mammalian radiation that commenced ~90–100 million years ago in the Cretaceous [55,56] or the origin of land plants more than 500 million years ago [49], which comprise the primary focus of our critique of summary coalescence methods in previous work (e.g., [7,13,53,57]).

By contrast with Lanier and Knowles’ [20] simulation study with eight taxa and just 1–9 loci (1000 bp each), Song et al.’s [27] mammalian data set included 36 mammals and 447 loci with an average gene length that spans 139.6 kb from start codon to stop codon. Song et al. [27] analyzed their data with the summary coalescence methods STAR [8] and MP-EST [9], not *BEAST or STEM. Whereas Lanier and Knowles’ [20] estimated that as many as ~17 recombination events can be detected with eight taxa and 1000 bp, Hobolth et al.’s [18] empirical estimates for recombination breakpoints in hominids suggest there are ~1358 recombination events for a typical 139.6 kb fragment with three taxa and an outgroup [7]. When Hobolth et al.’s [18] recombination rate is extended to Song et al.’s [27] data set with 37 taxa and ~9 difficult to resolve internodes that are similar to the human-chimp-gorilla clade, there are ~12,222 recombination events at ~11,760 different recombination breakpoints for a 139.6 kb segment of DNA, the vast majority (>90%) of which will affect topology (i.e., deep coalescence) and not just branch length heterogeneity because Hobolth et al. [18] ignored all forms of the latter except for the contrast between HC1 (human + chimp without deep coalescence) and HC2 (human + chimp with deep coalescence) (see Table 1 in Springer and Gatesy [7] for calculations).

This large discrepancy between the estimated number of recombination events for Song et al.’s [27] empirical data and Lanier and Knowles’ [20] simulated data suggests that the latter cannot be used to argue that intralocus recombination is not a problem for the former. Even if we use Munch et al.’s [38] recombination rate, which is approximately 19-fold lower than for Hobolth et al. [18], there are 12,222/19 = 643.2 recombination events for a typical 139.6 kb segment of DNA with taxonomic diversity similar to that of Song et al.’s [27] data set. Future simulations should match the empirical problem as closely as possible, including recombination rate, number of taxa, number of loci, phylogenetic depth, missing data, the absence of a molecular clock (if appropriate), and the choice of methods that are used to analyze the data (e.g., *BEAST [58] versus STAR [8]). We are unaware of any coalescence simulations studies with recombination that are comparable to Song et al.’s [27] mammalian data set and conclude that Lanier and Knowles’ [20] simulations are not relevant for interpreting Song et al.’s [27] data in the face of recombination rates suggested by Hobolth et al. [18,28] and Munch et al. [38] for several hominids, all of which are included in Song et al.’s [27] mammalian data set.

Finally, and also of critical importance, it is paradoxical that Lanier and Knowles’ [20] concluded that species tree estimation under the multispecies coalescent is robust to intralocus recombination when the dancing partner of recombination, ILS, is the raison d’etre for coalescence methods. That is, widespread intralocus recombination is required to generate the large number of independently segregating c-genes that are necessary to infer an accurate species tree in difficult circumstances such as the anomaly zone. For species trees in the anomaly zone, there should be a tendency for the true species tree inferred from correctly delineated c-genes (and accurately reconstructed gene tree topologies) without intralocus recombination to degrade to an inaccurately reconstructed species tree when individual c-genes are amalgamated into longer, pseudo c-genes that incorporate increasing levels of intralocus recombination. This argument follows logically from the multispecies coalescent, because concatenation of all genes in a dataset represents the most extreme case of intralocus recombination (all c-genes merged into one giant pseudo c-gene) and is expected to fail in the anomaly zone. A different interpretation of Lanier and Knowles’ [20] results is that intralocus recombination does impact species tree accuracy, but that any negative effects of recombination in Lanier and Knowles’ [20] simulations are dwarfed by the negative effects of gene tree reconstruction error and the extremely limited number of genes in each simulated data set. In these simulations, species tree methods may not have been powerful enough to detect any ill effects of recombination given that only 1–9 simulated genes were analyzed. For example, in the most challenging regions of tree-space that were explored, a relatively deep species tree with a rapid radiation of lineages, the inferred coalescence species tree was highly inaccurate with or without recombination. If the species tree methods employed by Lanier and Knowles [20] are powerful enough to infer the correct species tree for difficult problems in the anomaly zone, where concatenation is expected to fail, then there should be a decreasing probability of inferring the correct species tree with longer loci that include more recombination breakpoints, because ignoring intralocus recombination is the functional equivalent of concatenation. Stated differently, recombination must matter at some scale if coalescence methods effectively address the problem of ILS.

In another relevant simulation study, Wang and Liu [21] compared data-driven methods for detecting recombination and reported that pipelines that use direct analysis of the data to explicitly infer breakpoints and delineate c-genes (‘recombination-free intervals’) result in greater species tree accuracy with the summary coalescence method ASTRAL II relative to pipelines that pre-process loci into intervals of a given length based on independent estimates of linkage disequilibrium decay. Wang and Liu’s [21] explicit recombination approach was limited to detecting recombination breakpoints with the four-gamete test (FGT) [59]. The FGT assumes the infinite sites model, so all violations of the four-gametes rule are interpreted as recombination. This model is increasingly likely to conflate homoplasy with recombination at higher sequence divergence, but is also conservative and may underestimate recombination relative to models such as CoalHMM because only breakpoints associated with topological differences that are supported by underlying parsimony informative characters can be detected with the FGT. Still, Wang and Liu’s [21] study is important because it implies that “recombination is a problem for widely used approaches to species tree analysis” and should be addressed with methods that explicitly infer recombination breakpoints. Wang and Liu’s [21] implementation of the FGT showed that at shallow divergences (i.e., tree depth = 1 N) this approach can improve estimation of species tree topology relative to approaches that do not explicitly account for recombination breakpoints, However, as for Lanier and Knowles [20], no comparisons to concatenation were made, and deep divergences in the Tree of Life were not simulated. Therefore, it remains unclear how summary coalescence methods compare to concatenation when intralocus recombination is modeled with high sequence divergence.

## 7. Comparison of Different Recombination Breakpoint Methods

We performed recombination detection analyses with FGT for four target regions from Hobolth et al. [18] and Dutheil et al. [31] for human, chimp, gorilla, and orangutan. The FGT is typically employed within or between populations, but can also be used for recently diverged species [21,60,61]. The FGT was performed with the RminCutter.pl v1.05 (https://github.com/RILAB/rmin_cut/) python script (Ibarra) and the results of these analyses were compared to Hobolth et al.’s [18] CoalHMM results for the same target genomic regions. For each of the four targets, CoalHMM resulted in more recombination breakpoints and smaller c-genes than the FGT, suggesting that the FGT may be conservative relative to CoalHMM for this data set (Table 1). This result is expected given that CoalHMM is a model-based Bayesian approach that takes branch lengths into account and recognizes separate c-genes for segments of DNA that support human + chimp without deep coalescence (HC1) and human + chimp with deep coalescence (HC2). Nevertheless, even the FGT suggests that c-genes are typically much smaller than the complete protein-coding sequences that are routinely employed in recombination-ignorant coalescence analyses. Mean FGT c-genes based on the four target regions of Hobolth et al. [18] range from 726.3 bp for target 122 to 1097.5 bp for target 1 (Table 1). For the FGT results, c-genes are longer for human + chimp than for the alternative c-genes (human + gorilla, chimp + gorilla) that require deep coalescence. These results are consistent with those of CoalHMM [18] for the same target regions.

**Figure 2:**
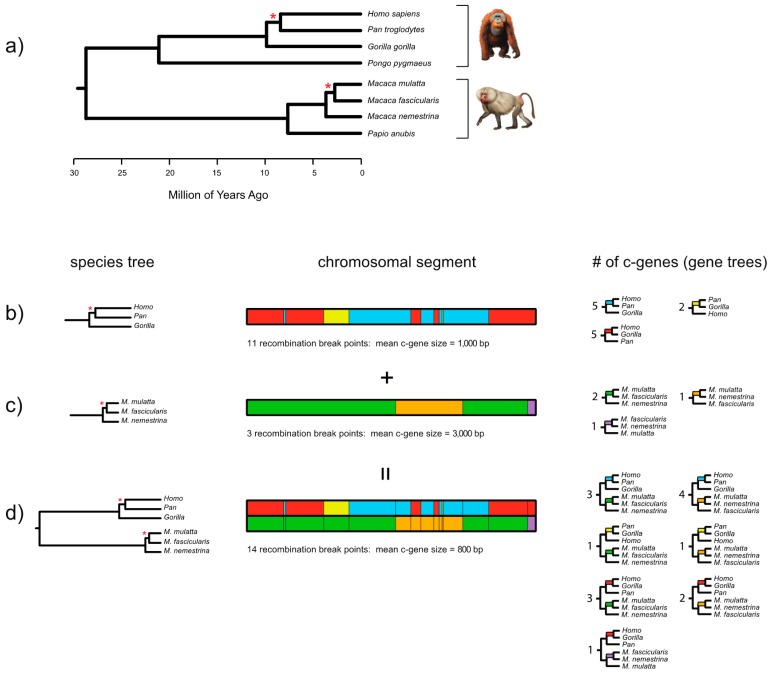
Results of four-gamete test (FGT) for the *ARMC3* gene (~120.1 kb alignment). The FGT was applied separately to two subtrees of primates (panel **a**)—four hominids (*Homo, Pan, Gorilla, Pongo*) and four cercopithecids (3 *Macaca* spp., *Papio*). Primate relationships are as in Springer et al. [71]. The FGT was applied to the entire *ARMC3* gene from start codon to stop codon, but the results are only illustrated for the first 12 kb of the alignment. Recombination breakpoints and c-gene trees are shown for hominids (panel **b**) and *Macaca* spp. (panel **c**). In both cases there are three possible topologies, all of which are represented by one or more c-gene trees (the outgroups *Pongo* and *Papio* are not shown). Panel (**d**) shows the results of the recombination ratchet, where overlay of 11 recombination breakpoints for hominids and three recombination breakpoints for *Macaca* results in 14 recombination breakpoints and 15 c-genes for the 12 kb alignment (panel **d**). Among the nine topologies that are possible for the two subtrees of three taxa, seven are represented among the 15 c-gene trees (panel **d**). Paintings of *Pongo* and *Papio* by Carl Buell.

Figure 2 shows the results of applying the FGT to complete gene sequences for *ARMC3*, which is one of Song et al.’s [27] protein-coding loci. In this case, we aligned the gene sequences from start codon to stop codon, including the intervening introns, for eight primates. The total alignment comprised 120,075 bp for eight primates that index two short branches (one in human-chimp-gorilla subclade, one in *Macaca* subclade). Based on this alignment the FGT was applied separately to two groups of four taxa (human, chimp, and gorilla with an orangutan outgroup; three species of macaques [*Macaca*] with a baboon [*Papio*] outgroup). Application of the FGT to the human-chimp-gorilla subclade resulted in 110 recombination breakpoints and 111 c-genes, whereas application of the FGT to the macaque subclade resulted in 23 recombination breakpoints and 24 c-genes. The recombination ratchet therefore suggests that there are 134 detectable c-genes for the full set of eight taxa (Figure 2). After excluding intronic c-genes, 15 different c-genes are represented by the 18 exons as follows: exon 1, exon 2, exon 3, exons 4 + 5, exon 6, exon 7, exons 8 + 9, exon 10, exon 11, exon 12, exon 13, exons 14 + 15, exon 16, exon 17, and exon 18. By contrast with the 110 recombination breakpoints that were detected with the FGT for the human-chimp-gorilla subclade based on complete gene sequences (exons and introns from start codon to stop codon), application of this test to an alignment with just the protein-coding exons for these three taxa (2619 bp) resulted in the detection of only one recombination breakpoint and two c-genes. This result underscores the importance of using contiguous sequences, including the intervening introns, when recombination breakpoint methods are applied to complete protein coding sequences.

We also analyzed Hobolth et al.’s [18] target regions with seven different recombination detection programs that are implemented in the program RDP4 [62]. These methods are RDP [63], GENECONV [64,65], BootScan [66], MaxChi [67], Chimaera [68], SiScan [69], and 3Seq [70]. All of these methods detected far fewer recombination breakpoints than CoalHMM and FGT (Table 2). Among the seven methods, RDP detected the largest number of recombination breakpoints (total = 90 for four target regions) and SiScan detected the fewest recombination breakpoints (total = 5 for four target regions). These results are perhaps not surprising given that some of these methods were not designed specifically to detect ILS. For example, GENECONV was developed to detect gene conversion [64] and MAXCHI [67] was developed to detect horizontal gene transfer in prokaryotes. In such scenarios, long runs of conflicting characters that support different topologies can simplify detection of evolutionary events. By contrast, some cases of ILS may be associated with nothing more than branch length heterogeneity [7,26]. Our results are consistent with the simulation study of Posada and Crandall [68], who concluded that many of these methods (GENECONV, MaxChi, RDP, Chimaera, BootScan) are not very powerful.

## 8. Binning and Transcriptome Data

Warnow and colleagues [72,73,74] have developed “binning” methods that combine loci from different regions of the genome prior to the estimation of supergene trees from the binned (concatenated) loci that serve as input for species tree reconstruction using coalescence methods. Liu et al. [22] have previously criticized binning methods that combine c-genes from different regions of the genome: “Ideally, loci should be concatenated if no or only a few recombination events occurred between those loci. A model based on biology would suggest that binning should be based on loci that are closely linked in genomes, such as often occurs in transcriptomes, because the chance of recombination is positively related to the physical distance between two loci.” We suggest that a model based on biology (e.g., [18,38]) will also preclude, in many instances, concatenation of exons from the same protein-coding gene because these exons will have histories that are just as different as exons from different genes [19]. Also consider a simple problem with three ingroup taxa (human, chimp, gorilla) and one outgroup. Gene trees associated with adjacent c-genes (ignoring branch length heterogeneity) will always have a normalized RF distance of 1. Similarly, the RF distance for conflicting gene trees from far-flung regions of the genome will always equal 1. Thus, in this case it is no more appropriate to combine conflicting c-genes that are adjacent than it is to combine c-genes from different chromosomes. With more taxa, the genealogical histories of nearby c-genes will be autocorrelated and will be more similar to each other, on average, than to randomly sampled c-genes from different chromosomes (Figure 1). However, c-genes will also be smaller with more taxa because of the recombination ratchet. Consider a case with six taxa and two trichotomies that are similar to the human-chimp-gorilla problem. The maximum RF distance between different gene trees will equal 2 if topological variation is restricted to the two trichotomies. RF distances of 2 are only possible when c-genes are non-adjacent except in cases where recombination breakpoints are convergent (occur at the exact same chromosomal position) in each of the separate three-taxon clades. More commonly, RF distances of 1 will describe genealogies for adjacent c-genes. Also, individual c-genes for six taxa and two trichotomies will be, on average, ~0.5× the size of c-genes with a single trichotomy if equivalent levels of ILS occur in each subclade as in Figure 1. In such cases maximum RF distances can still be achieved over very short distances, i.e., two recombination breakpoints. If c-genes are ~12 bp for a data set similar to that of Song et al. [27] with 36 mammalian taxa, then terminal exons will be separated by ~11,760 recombination breakpoints for a locus that spans 139.6 kb from start codon to stop codon (mean value for Song et al.’s [27] loci). If topological variation due to ILS/recombination is essentially restricted to nine trichotomies, each of which is represented by approximately the same number of gene trees, the maximum RF distance [sensu 25] will be 9 and the mean RF distance between randomly selected gene trees will be ~6 (i.e., random gene trees will differ at six of nine trichotomies if each of the three resolutions for each trichotomy is equally likely). C-genes and genealogies with RF = 6 can occur over intervals with as few as six recombination breakpoints, although with reversals at the same trichotomy this number can be be higher than 6. Nevertheless, this number of recombination events is orders of magnitude smaller than the number of recombination events that are predicted to occur for a 139.6 kb segment with nine trichotomies and recombination rates based on Hobolth et al.’s [18] estimates of this parameter. Even with Munch et al.’s [38] lower recombination rates, there are more than 600 recombination breakpoints for a 139.6 kb locus in mammals. We therefore contend that binned exons from the same protein-coding locus (e.g., transcriptome data) may often be just as divergent as c-genes that are randomly selected from different chromosomes. This implies that Liu et al.’s [22] criticisms of binning also apply to their own use of transcriptome data and complete protein-coding sequences in summary coalescence analyses.

## 9. Conclusions

Coalescence methods have emerged as a popular alternative for inferring species trees from DNA sequence data for multiple loci. Unlike fully parametric methods such as *BEAST [58], which are computationally intensive and can only be used with small or moderately large data sets, summary coalescence methods such as STAR [8], NJst [10], and ASTRAL-II [11] employ computational shortcuts and are routinely applied to large, genomic data sets with many taxa. By contrast with concatenation methods for species tree construction, summary coalescence methods explicitly address the problem of incomplete lineage sorting, which may compromise the accuracy of concatenation in the anomaly zone. However, summary coalescence methods make their own assumptions including no intralocus recombination within coalescence genes (c-genes), which are the basic units of analysis for these methods. Summary coalescence methods also assume accurate reconstruction of gene trees and thus accurate gene tree stoichiometry. Accurate gene tree stoichiometry implies that gene trees are sampled in proportion to their expected frequencies under the multispecies coalescent with complete neutrality—another assumption that is unlikely to hold in empirical data sets but was specified in simulations that test the accuracy of summary coalescence methods [7,53]. Violations of these assumptions may cause more problems for summary coalescence methods than failure to address ILS causes for concatenation, especially at deep levels in the Tree of Life where gene tree accuracy is impacted by long branches, inequalities in evolutionary rates, extensive homoplasy, and lack of phylogenetic signal at rapid radiations [7,53]. Advocates of summary coalescence approaches have routinely employed complete protein-coding sequences to reduce problems with gene tree inaccuracy, but in taxa such as mammals, complete protein-coding sequences typically include multiple exons and can span intervals that are much longer than individual c-genes. This problem is compounded by the recombination ratchet, which describes the inverse relationship between c-gene size and increased taxon sampling in a dataset (Figure 1 and Figure 2). Advocates of summary coalescence methods (e.g., [15]) have also argued that these methods are robust to intralocus recombination based on limited simulations [20]. However, these simulations were performed at relatively shallow depths in the Tree of Life with a maximum of only nine loci and no comparison to concatenation for the same data sets. These simulations therefore are of little relevance for deeper divergences such as the placental radiation and the diversification of the major lineages of angiosperms where phylogenomic datasets of hundreds or thousands of loci have been applied for many more taxa. Most importantly, recombination must matter at some scale because the direct product of recombination, ILS and associated shifts in gene tree topologies across the genome, are the primary reasons for the development of coalescence methods for species tree inference. Given that the end member (i.e., extreme case) of increased locus length is concatenation, how long is too long for loci that include recombination and are employed in coalescence analyses? Liu et al. [22] suggested that binning of exons from the same gene is a biologically justified model, but Scornavacca and Galtier’s [19] results for mammals suggest that exons from the same gene are just as different as exons from different genes that are spread across the genome. Exons within mammalian genes should therefore not be combined in coalescence analyses unless appropriately sensitive tests for recombination suggest that adjacent exons share the same history. Future studies with summary coalescence methods should pay more attention to the proper demarcation of c-genes given that these are the fundamental units of analysis for these methods.

## Figures and Tables

**Table 1:** Comparison of the number of recombination breakpoints and coalescence genes (c-genes) based on CoalHMM and the four-gamete test (FGT) for four target regions from Hobolth et al. [18].

Target Number	Target Length	Mean CoalHMM C-Gene Length	Four-Gamete Test		
			Number of Recombination Breakpoints	Number of C-Genes	Mean FGT C-Gene Length
1	1255492	123.0	1143	1144	1097.5
106	257420	84.1	303	304	846.8
121	230666	102.9	287	288	800.9
122	92240	108.8	126	127	726.3

CoalHMM c-gene sizes are based on Hobolth et al.’s [18] analyses with additional calculations reported in Springer and Gatesy [7].

**Table 2:** Comparison of the number of recombination breakpoints and mean c-gene size based on seven different recombination detection methods implemented in RDP4 [62] that were applied to four target regions from Hobolth et al. [18].

Method	Number of Recombination Breakpoints/Mean C-Gene Length (kb)			
	Target 1	Target 106	Target 121	Target 122
RDP	45/27.3	21/11.7	20/11.0	4/18.4
GENECONV	29/41.8	11/21.5	9/23.1	5/15.4
MaxChi	17/69.7	4/51.5	7/28.8	4/18.4
Chimaera	14/83.7	1/128.7	8/25.6	2/30.7
BootScan	36/33.9	14/17.2	16/13.6	9/9.2
3Seq	26/46.5	9/25.7	8/25.6	2/30.7
SiScan	4/251.1	1/128.7	0/230.7	0/92.2

For each method and target region, the number of recombination breakpoints is shown before the slash and mean c-gene size (in kb) is shown after the slash.
